# Spanish West Nile virus isolates of lineages 1 and 2 elicit different patterns of infection in red-legged partridges

**DOI:** 10.1099/jgv.0.002186

**Published:** 2025-12-04

**Authors:** Rafael Gutiérrez-López, Raúl Fernández-Delgado, Pilar Aguilera-Sepúlveda, Desirée Dafouz-Bustos, Miguel Ángel Jiménez-Clavero, Francisco Llorente

**Affiliations:** 1 Centro de Investigación en Sanidad Animal (CISA-INIA), CSIC, Valdeolmos, Spain; 2 CIBER en Epidemiología y Salud Pública, CIBERESP, Madrid, Spain

**Keywords:** arbovirus, emerging infectious diseases, flavivirus, host competence, vector-borne diseases

## Abstract

During the summer of 2020, West Nile virus (WNV) caused an important outbreak in south-western Spain, with the highest impact on humans in the country up to that time, resulting in 77 clinical cases (including eight fatalities). Concurrently, equine WNV foci were reported within the same region. Meanwhile, WNV circulation was also detected in horses and birds in north-eastern Spain (Catalonia), although no human cases were notified. This striking difference in human case incidence between these two affected areas may be due to characteristics of the strains circulating in each site. One of these intrinsic viral strain factors that may account for these differences is the competence of avian reservoir hosts. A higher host competence leads to a higher viral spread in the enzootic cycle, consequently, increasing the risk of spillover to humans and horses. To assess differences in host competence, WNV strains circulating in both areas during the summer of 2020 were studied through *in vivo* inoculation of a transmission-competent avian species susceptible to WNV infection, the red-legged partridge, autochthonous to the Iberian Peninsula. The south-western strain SPA20-02, belonging to lineage 1, and the north-eastern strain AC924, belonging to lineage 2, were inoculated in parallel in red-legged partridges. The SPA20-02 strain exhibited higher and longer viraemias than the AC924 strain, resulting in a higher competence index for this avian species. The lower competence index of red-legged partridges for transmission of AC924 suggests that this strain exhibits a lower transmission capacity and, consequently, lower spread risk. These findings indicate that the lower severity of the 2020 outbreak in north-eastern Spain could, at least partially, be explained by the reduced transmission potential of the AC924 strain.

## Introduction

West Nile virus (WNV, family *Flaviviridae*, genus *Orthoflavivirus*) is a widely distributed zoonotic, mosquito-borne virus. Its life cycle involves multiple bird species as amplifying hosts, while the main transmission vectors are different species of mosquitoes in the *Culex* genus [1,3]. Horses and humans are susceptible to infection; however, they are not competent hosts, as they do not develop sufficiently high viraemia to transmit the virus to mosquitoes that feed on their blood [4].

In humans, the infection is often asymptomatic or results in mild clinical signs described as West Nile fever (WNF). However, less than 1% of infected individuals may develop severe neurological diseases, including encephalitis or meningoencephalitis, which can be fatal [5,6]. WNV was first identified in the blood of a febrile woman from the West Nile district (Uganda) in 1937 [7]. The virus has significantly expanded from Africa to other regions of the world, especially during the past three decades. Nowadays, it is considered one of the most widespread arboviruses globally, having been detected in Africa, Europe, the Middle East, Asia, Oceania and the Americas [8,10].

Reports of WNV infection in Europe date back to the 1960s [11]; since then, numerous cases in humans and equines have been reported, with a significant increase in detections since the 1990s [8]. In Europe, WNV circulates across several countries, where it is considered endemic, and it is expanding its geographical range into northern areas [12]. Prior to 2010, WNV outbreaks in Europe were predominantly caused by lineage 1 (L1) strains [8,13]. However, this changed after the introduction of lineage 2 (L2) into the continent in 2004 [14] and its subsequent spread, particularly since 2010. This was evident when large outbreaks affected Greece and the Balkans, from where the virus expanded to other parts of Europe [13,15,17]. In addition, WNV L2 replaced L1 in some regions, such as northern Italy, where it remained the predominant strain during an 8-year period [18].

In Spain, the first evidence of WNV circulation dates back to 2003, although it was likely present prior to this date [19,20]. However, it was first isolated from vertebrate hosts in 2007, specifically from two golden eagles (*Aquila chrysaetos*) [21]. Cases of West Nile neurological disease in horses and humans were subsequently reported in 2010 in southern Spain (Andalusia), caused by a Ll strain [22]. Since then, WNV has become endemic in south-western Spain, causing outbreaks annually in horses and sporadically in humans, e.g. three confirmed cases in 2016 [23], all of them involving L1 WNV. In September 2017, WNV was detected on the opposite side of the country, in Catalonia (north-eastern Spain), ~700 km away from the south-western foci, through passive surveillance in Eurasian goshawks (*Accipiter gentilis*) [24]. Phylogenetic analyses revealed its relation to the Central/Southern European clade, indicating the spread of WNV L2 towards Western Europe [15].

In the summer of 2020, the first severe outbreak of WNV in Spain occurred in south-western Spain (affecting two Autonomous Communities: Andalusia and Extremadura), causing 77 clinical human cases (including eight fatalities) [25]. Numerous equine WNV foci were also reported in these regions at the same time. The outbreak was also correlated with a higher prevalence of seroconversion in horses compared with previous years [26]. Additionally, a geographical correlation between the incidence of human cases and seroconversion in birds was detected [27]. All WNV strains detected and isolated in the area during 2020 and previous years were identified as L1 [28]. More recently, in 2024, a new outbreak affected Andalusia and Extremadura, accounting for 158 human cases, 20 of them fatal, as well as numerous avian and equine cases [29,30]. This outbreak was still caused mostly by L1 strains, though a first incursion of L2 in Andalusia was reported in 2024 in the Jaén province [31]. Meanwhile, in Catalonia, although no human cases were reported during 2020, new isolations of L2 were obtained from raptors [15], demonstrating the active circulation of this recently introduced lineage in that territory.

The spread of both WNV lineages to new regions highlights the virus’s ability to spread beyond its traditional viral circulation niches and its adaptation to local hosts and vectors. Infections in wild birds have been observed, affecting species such as the Eurasian goshawk [15], snowy owl (*Bubo scandiacus*), Chinese merganser (*Mergus squamatus*), black-tailed gull (*Larus crassirostris*), great tit (*Parus major*) [32], griffon vulture (*Gyps fulvus*) and the little owl (*Athene noctua*) [33,34]. A wealth of information exists regarding WNV experimental infections in birds, particularly with the NY99 (L1) strain [35,43]. One of the causes that may explain the increase in outbreaks is the emergence of newly evolved strains better adapted to reach higher competence for transmission to vectors in local hosts. In this regard, studies on the course of infection by Euro-Mediterranean strains in different avian species, as well as the role that native bird species play in the spillover of these strains, remain scarce. Therefore, the analysis of the course of the infection (including pathogenicity and host competence) of newly emerged strains in native avian species potentially acting as reservoirs merits attention for enhancing the understanding of WNV eco-epidemiology.

In this regard, the native red-legged partridge (*Alectoris rufa*) populations may play a relevant role in the epidemiology of WNV in Spain. Serological evidence supports the circulation of WNV in this bird species [44]. In addition, it is a suitable avian model for flavivirus experimental research [42,45, 46]. Notably, it has been found to reach viraemic levels high enough to infect mosquitoes, thus acting as a competent host for the virus. Furthermore, it has been proven to be a useful model for discriminating between WNV strains based on pathogenicity and host competence [42,47, 48].

The WNV outbreak of serious public health consequences affecting Andalusia and Extremadura in 2020 contrasted with the almost silent circulation of WNV in Catalonia during the same transmission season. These differences in severity could be attributed to the phenotypic characteristics of each strain involved, including their pathogenicity, transmissibility or other phenotypic traits. Hence, the main objective of this study was to assess the course of infection elicited by *in vivo* inoculation of red-legged partridges with WNV strains isolated from the Andalusian and Catalonian outbreaks, including the evaluation of pathogenicity and host competence in this avian host.

## Methods

### Viruses and virus preparations

The strains analysed in this study were as follows: SPA20-02 (L1), from south-western Spain (Andalusia), and AC924 (L2), from north-eastern Spain (Catalonia). In addition, the strain It´08 (L1), from Italy, previously characterized in red-legged partridges [48], was used as a positive control for the assay. Details of their origin and number of cell passages are indicated in Table 1. All the strains were titrated by plaque assay in Vero cells, and virus titres were expressed in p.f.u. [49].

**Table 1.** Viral strains used in this study, origins, number of passages in cell culture and source information

Virus strain	Geographic origin	Species	Year of isolation	Cell passage no.	GenBank accession no.	Source of the strain
SPA-E-2020/02 (SPA20-02)	Spain (Andalusia, Seville)	Horse (*Equus caballus*)	2020	3pVR	OP713602	Laboratorio Central de Veterinaria (LCV-MAPA)
AC924	Spain (Catalonia, Tarragona)	Goshawk (*A. gentilis*)	2020	2pVR	OM037673	Centre de Recerca en Sanitat Animal (IRTA-CReSA)
Italy15803/08 (It´08)	Italy (Brescia)	Magpie (*Pica pica*)	2008	3pVR	FJ483549	Istituto Zooprofilattico Sperimentale della Lombardia e dell’Emilia Romagna (IZSLER)

VR: Vero cells.

### Red-legged partridges

Six-week-old red-legged partridges (*A. rufa*) (*n*=70) were kindly provided by the Lugar Nuevo breeding facility (Estación de Referencia de la Perdiz Roja, Consejería de Medio Ambiente y Ordenación del Territorio-Junta de Andalucía, Andújar, Spain). The partridges were transported to the biosafety level 3 (BSL-3) facilities at CISA (Centro de Investigación en Sanidad Animal, Valdeolmos, Spain), where they were housed in four wire-mesh cages (three cages containing 19 birds each, and one cage with 13) after external deparasitation. During the experiment, the partridges had *ad libitum* access to water and a commercial diet formulated for game birds. A blood sample (0.1–0.2 ml) and an immature feather were collected from each individual to discard previous exposure to WNV serologically, by using a commercially available competitive ELISA (INgezim West Nile Compac; Gold Standard Diagnostics, Madrid, Spain) and virologically by real-time reverse transcription PCR (RT-PCR) [50].

### Experimental procedure and sample collection

**Fig. 1.**
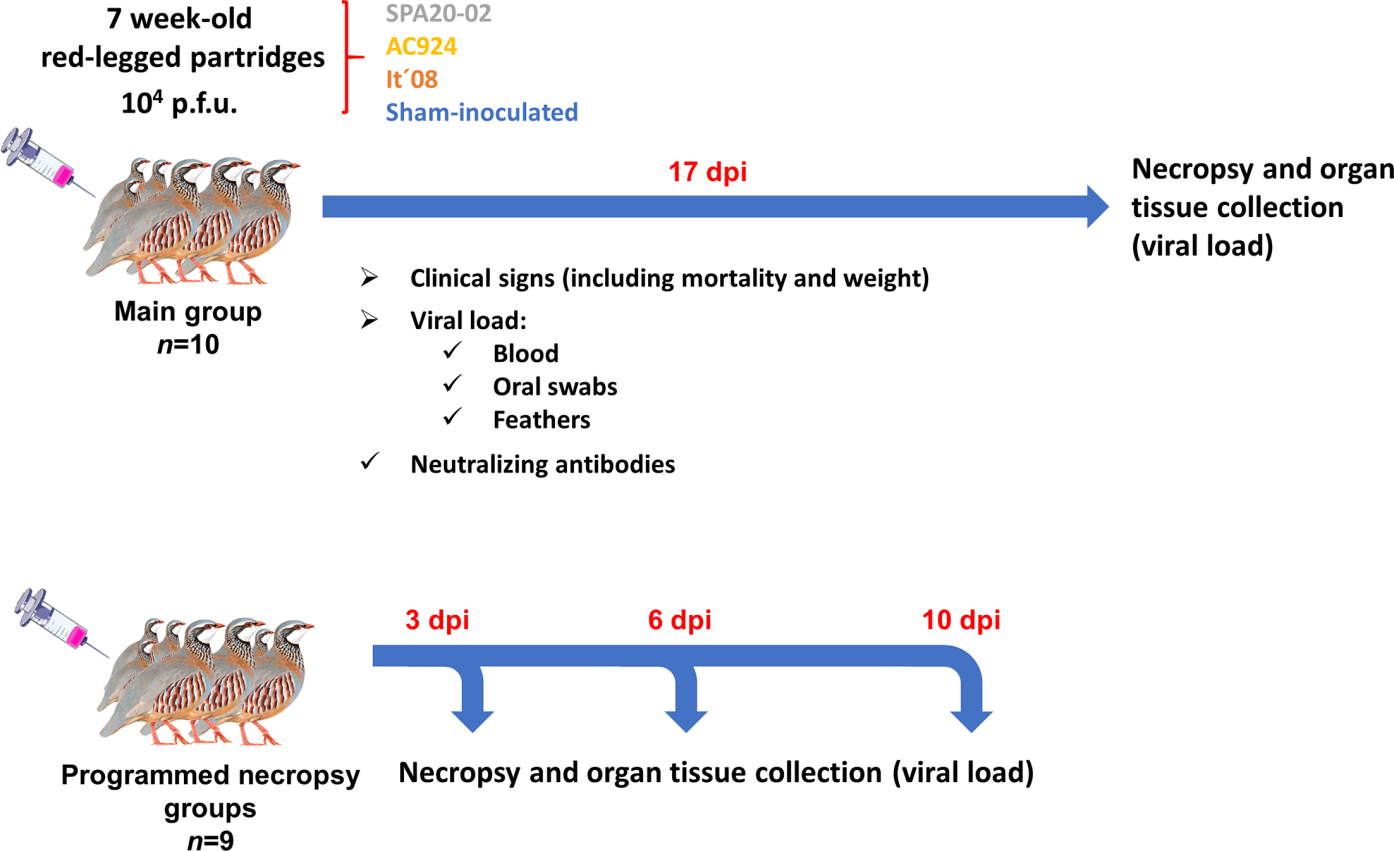
Schematic representation of the experimental infection procedure in the red-legged partridges.

Following a 7-day acclimatization period, three groups, each composed of nineteen 7-week-old red-legged partridges, were subcutaneously inoculated in the neck (10^4^ p.f.u. ml^−1^ per individual) with either the SPA20-02, AC924 or It´08 WNV strains (Fig. 1). The inocula (100 µl) were diluted in PBS containing 0.2% BSA. A negative control group (*n*=13) was sham-inoculated with an equivalent volume of diluent (from here named sham-inoculated group). Clinical signs were monitored daily, including changes in body weight for each individual, aspect of feathers, mobility alterations and other behavioural changes, for up to 17 days post-inoculation (dpi). A group of ten animals from each inoculated group was selected for weight determination and periodic sampling. Weights were measured at 0, 3, 5, 7, 9, 11, 14 and 17 dpi. Blood samples (0.1–0.2 ml per individual) were obtained from the jugular vein. To assess the course of viraemia, 0.1 ml of blood collected at 1, 3, 5, 7 and 9 dpi was immediately added to 0.9 ml of BA-1 diluent (Hanks M-199 salts, 0.05M Tris,pH 7.6, 1% BSA, 0.35 g/l of sodium bicarbonate, 100 U/ml of penicillin,100 μg/ml of streptomycin, 1 μg/ml of amphotericin B) in sterile polypropylene tubes. For antibody detection, blood (0.1–0.2 ml per individual) samples obtained at 1, 3, 5, 7, 9, 11, 14 and 17 dpi were collected in dry tubes and allowed to clot at 37 °C for 1 h, followed by overnight incubation at 4 °C to obtain serum. Oropharyngeal swabs were collected at 1, 3, 5, 7, 9, 11 and 14 dpi and placed in sterile polypropylene tubes containing 1 ml of PBS. Immature rump feathers were collected at 1, 3, 5, 7, 9, 14 and 17 dpi and collected in empty sterile polypropylene tubes. All samples were stored at −80 °C until analysis. The sham-inoculated group was handled, sampled and analysed in parallel, exactly in the same way as the inoculated groups. The other nine birds from each inoculated group were assigned as programmed necropsy bird groups. The programmed necropsy birds were euthanized and necropsied at 3, 6 and 10 dpi (three birds from each group per day, except for the group It´08, where an individual died before the programmed necropsy day). Birds that succumbed to the infection were necropsied within 24 h after death. Animals were euthanized at a pre-mortem stage when they were severely diseased with irreversible clinical signs and were considered as having succumbed to the infection for the analyses. Additionally, five partridges surviving from each inoculated group and three from the sham-inoculated group were also necropsied at the end of the experiment (17 dpi). Upon necropsy, samples (~0.1 g) of brain, heart, kidney, spleen and liver were collected in tubes containing 0.9 ml of PBS for real-time RT-PCR analysis. Single-use scalpels and forceps were used to avoid cross-contamination. When animals arrived at the BSL-3 facilities, with 6 weeks of age, sexual dimorphism was not yet apparent. Therefore, group composition could not be based on sex. At the end of the experiment, the sex of the animals was determined during necropsy. The inoculated groups for sampling (*n*=10) included between two and four female birds.

### Virus detection assays

Viraemia was measured by standard plaque formation assays as previously described [49]. The viral genome was extracted following different preparation steps depending on the type of sample [40]. RNA was extracted from blood, organs, feathers and swabs using a BioSprint 15 workstation (QIAGEN) and was subjected to real-time RT-PCR, as previously described. Cycle threshold (Ct) values obtained with this method are directly correlated with RNA copy number (correlation coefficients higher than 0.99), and samples with Ct ≥40.0 were considered negative [50].

### Calculation of host competence index

The host competence index (Ci) estimates the average number of infectious vectors generated from each viraemic host. It is characteristic of each host species. A higher host competence capacity, indicated by a higher Ci value, reflects how efficient the host species is to transmit the virus to vectors, and hence it is directly related to transmissibility of a given arbovirus strain through a specific host. The Ci value is calculated based on the formula Ci = s×i×d [51], where ‘s’ represents susceptibility (the proportion of birds infected upon exposure), ‘i’ represents infectiousness (the proportion of exposed vectors that become infectious per day) and ‘d’ represents the duration of infectiousness (the number of days a bird maintains an infectious viraemia). Infectiousness (i) was estimated using a standard curve for infection of *Culex pipiens* based on viraemic titre [52]. For this calculation, the mean viraemic peak (mean of the maximum viraemia for each individual in the group) was used. The duration of infectious viraemia (d) was calculated by interpolation of the points crossing the value y = 10^5^ p.f.u. ml^−1^ in the viraemic curve, as only viraemia titres above this threshold are considered infectious for *C. pipiens* mosquitoes [52].

### Antibody detection assay

Virus-neutralizing antibodies were titrated by virus neutralization test (VNT) in 96-well microplates, as previously described [53]. Serum dilutions were assayed against the Eg-101 WNV strain (GenBank accession number AF260968).

### Statistical analyses

Differences in weight variations during the experiment (dependent variable) among infected groups (independent variable) were analysed using the Kruskal–Wallis test in R software 3.2.5 [54]. When significant differences were found, multiple group comparisons were evaluated using Dunn’s test with Holm correction [55]. In addition, differences in body weight loss among groups at specific dpi were assessed using pairwise t-tests, including viral strain as a fixed factor and body weight at day 0 as a covariate to control for baseline differences. Survival curves of each sampled bird were compared by Kaplan–Meier analysis and analysed by the log-rank test using IBM SPSS (IBM Corporation). Viral genome load (Ct-value) in blood, feathers, oral swabs, organs and viraemia levels determined by plaque assay (p.f.u.) were compared among infected groups using the Kruskal–Wallis test. Differences among groups at specific dpi were assessed using pairwise t-tests. When significant differences were detected, pairwise comparisons were performed using Dunn’s test with Holm correction.

## Results

### Pathogenicity for red-legged partridge

**Fig. 2.**
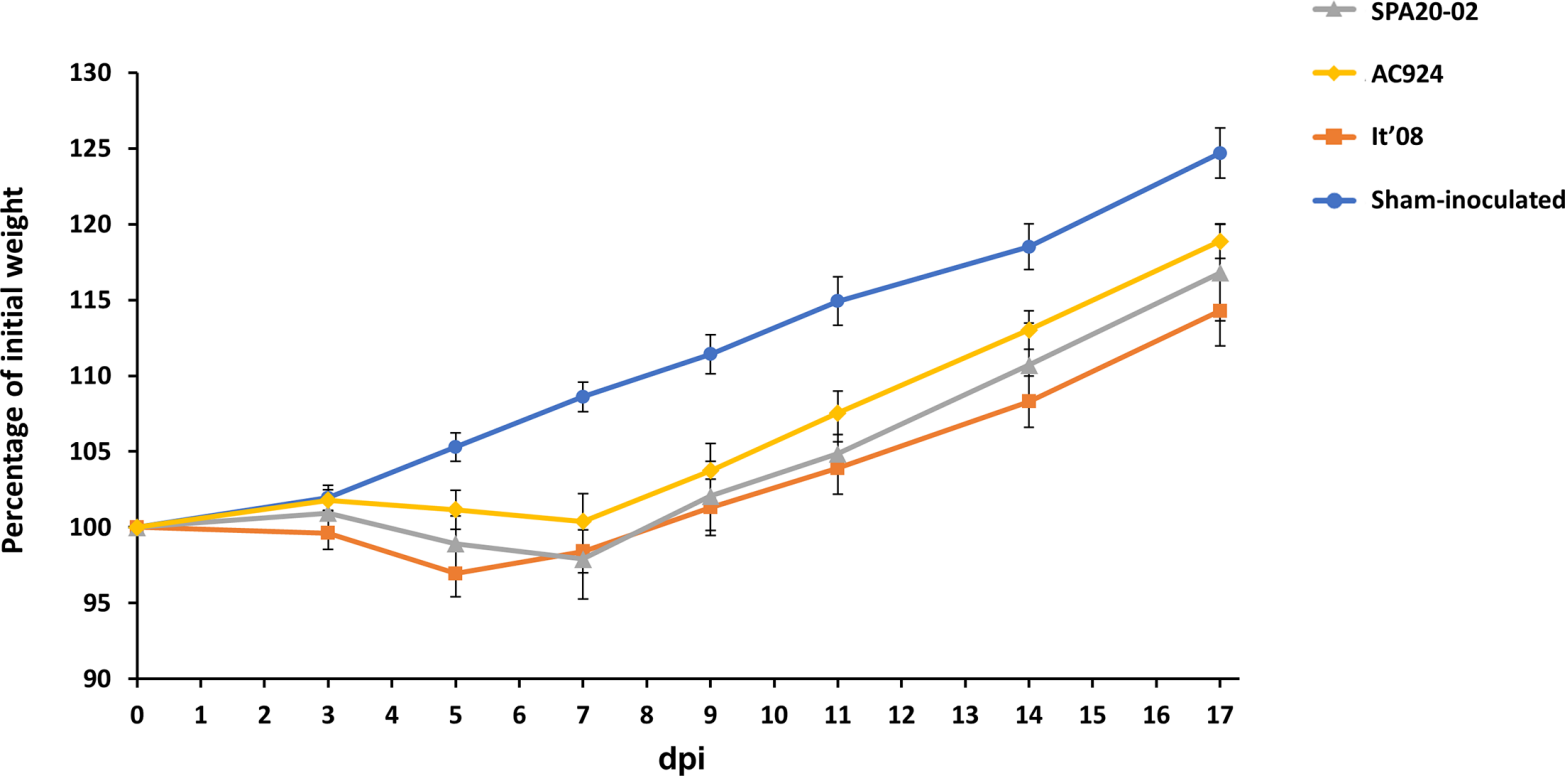
Weight curve of red-legged partridges after virus inoculation, expressed as the percentage of initial weight across different dpi. Bars represent the sem.

All WNV-inoculated partridges showed clinical signs (e.g. weight loss, ruffled feathers and lethargy) that started between 5 and 7 dpi. The sham-inoculated group stayed healthy and steadily gained weight during the experiment, as expected for the age of the birds (7-week-old at the day of inoculation). Weight gain throughout the experiment was significantly lower in the inoculated groups compared with the sham-inoculated group (SPA20-02 versus sham-inoculated group, *P*<0.01; AC924 versus sham-inoculated group, *P*=0.02; It´08 versus sham-inoculated group, *P*<0.01). However, no significant differences were observed in weight gain between the groups inoculated with SPA20-02 versus It´08 (*P*=0.46) or between those inoculated with AC924 versus SPA20-02 (*P*=0.14), although a significant difference was found when comparing the groups inoculated with It´08 versus AC924 (*P*=0.03) (Fig. 2). These differences in weight gain started to be noticed upon 5 dpi, when inoculated birds started to lose weight (Fig. 2). Between 7 and 9 dpi, virus-inoculated partridges regained weight, although the average weight gain was lower than in the sham-inoculated group by the end of the experiment (Fig. 2). A significant difference in body weight variation between It´08 and AC924 groups was detected at specific time points (5 dpi, *P*=0.03; 14 dpi, *P*=0.02; 17 dpi, *P*=0.04). Other unspecific clinical signs, including ruffled feathers and lethargy, were observed in most of the animals (>70%) inoculated with It´08 at 5 dpi, while in groups inoculated with the other strains, these signs appeared later (6–7 dpi) and in a lower proportion of animals (<30%). Severe clinical signs, including ataxia and leg paralysis, were detected only in a single partridge inoculated with SPA20-02 at 7 dpi. No differences in clinical signs were detected between males and females.

**Fig. 3.**
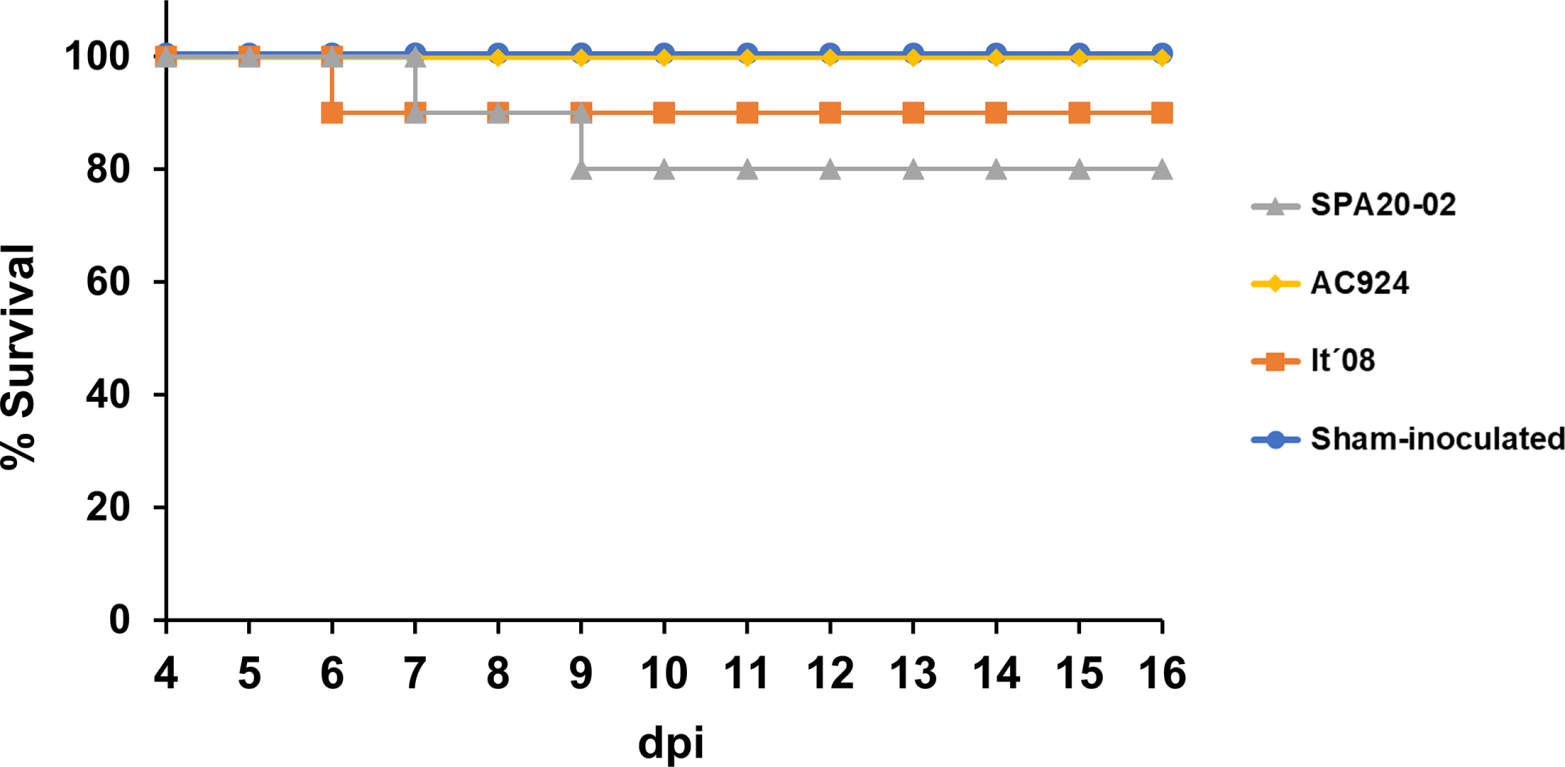
Survival curves in red-legged partridges after subcutaneous inoculation of 10^4^ p.f.u. of different WNV isolates, or sham-inoculated.

Regarding survival, non-significant differences in mortality rate between groups were detected (*P*>0.7 for all the comparisons). In sampling birds’ groups, two birds infected with SPA20-02 died at 6 and 9 dpi, and only one bird inoculated with It´08 died at 5 dpi. No deaths were detected in the individuals infected with AC924 nor in the sham-inoculated group during the experiment (Fig. 3).

The macroscopic examination of the birds included in programmed necropsy groups revealed no gross lesions at 3 dpi, whereas at 6 dpi, one bird inoculated with the It´08 strain developed thickened and opaque pericardium, consistent with pericarditis, and two additional birds exhibited congested intestinal blood vessels. Additionally, one of the two animals euthanized at 10 dpi presented vascular congestion in the intestine. In SPA20-02-infected birds, the only observed lesions were pallor of the hepatic parenchyma and injected intestinal blood vessels in two birds at 6 dpi, and pale areas in the liver in one bird at 10 dpi. In the AC924 programmed necropsy group, the only macroscopic alteration was vascular congestion in the intestine in one bird at 6 dpi. No lesions were observed in the animals euthanized at the end of the experiment (17 dpi). Macroscopic lesions were more frequently observed in animals that succumbed to the infection. In the It´08 group, the bird that died at 4 dpi showed only pale hepatic and renal parenchyma, but the animal that died at 7 dpi showed ascites, pale areas in the liver parenchyma and a distended gallbladder with very dark content. The bird that succumbed to the SPA20-02 infection at 7 dpi exhibited a very poor body condition, with complete absence of abdominal fat. The bird that died at 9 dpi showed ascites, pale areas in the hepatic parenchyma, splenic congestion and injected blood vessels in the intestine.

### Viral detection in blood, feathers, oral swabs and organs

**Fig. 4.**
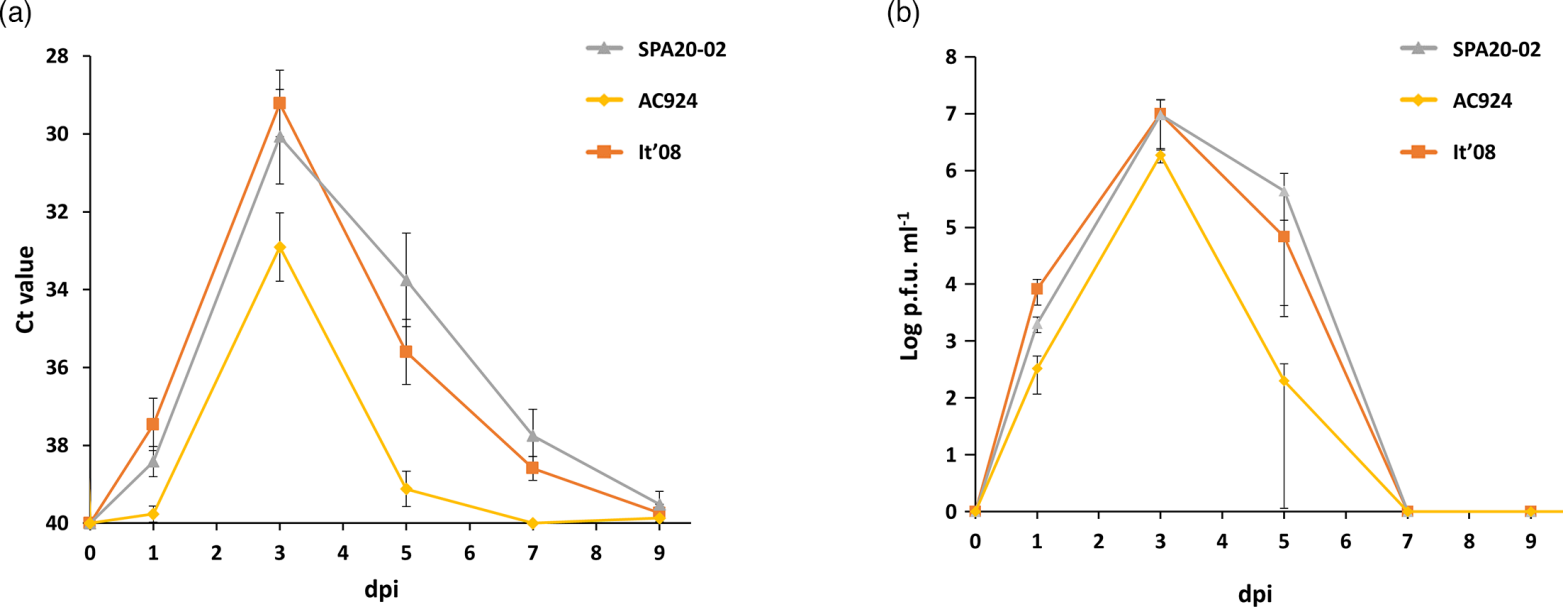
Mean daily viral genome load in blood (**a**) and viraemia titres (**b**) for the three inoculated groups. Each point represents the mean obtained for the surviving individuals at different dpi. Bars represent the sem.

All WNV-inoculated partridges developed detectable virus genome in their blood, except for one partridge inoculated with the AC924 strain whose blood remained negative throughout the whole experiment. In the three WNV-inoculated groups, viral RNA was detected at 1 dpi, with a peak at 3 dpi (Fig. 4a). The group infected with AC924 showed a significantly higher average Ct value (i.e. lower viral RNA load) throughout the experiment (between 1 and 7 dpi) compared with the groups infected with SPA20-02 (*P*<0.01) and It´08 (*P*<0.01). There were no significant differences in the average Ct values throughout the experiment between the groups infected with SPA20-02 and It´08 (*P*=0.95) (Fig. 4a).

All inoculated partridges developed detectable viraemia, as confirmed by plaque assay (except for one individual inoculated with AC924, also with undetectable viral RNA in blood). Overall, the viraemia peak was observed at 3 dpi. Although no statistically significant differences were observed between the whole viral-load curves of the different WNV-inoculated groups (χ²=5.12; degrees of freedom (d.f.)=2; *P*=0.08), significant differences in the viraemia were detected at specific time points between AC924 and the other inoculated groups: at 1 dpi (SPA20-02, *P*<0.01; It´08, *P*=0.01) and at 5 dpi (SPA20-02, *P*=0.02; It´08, *P*<0.01).

All groups of WNV-inoculated partridges, regardless of the strain used for inoculation, exceeded the established threshold of 10^5^ p.f.u. ml^−1^ in blood, indicating that this species is a competent host for the transmission of the three WNV strains analysed in this study. However, differences in the calculated Ci values were observed between the strains (Table 2). The group inoculated with AC924 showed a shorter period of infectious viraemia (higher than 10^5^ p.f.u. ml^−1^) than the groups infected with SPA20-02 and It´08 (Fig. 4b). This shorter period of infectiousness implies a lower host competence for AC924. Therefore, although the red-legged partridge is a competent host for the transmission of all WNV strains studied herein, its host competence is higher for SPA20-02 (Ci=0.73) and It´08 (Ci=0.69) when compared with AC924 (Ci=0.20) (Table 2).

**Table 2.** Host Ci for each inoculated group

Virus strain	Susceptibility (s)	Infectiousness (i)	Duration (d)	Competence index (Ci)
SPA20-02	1	0.22	3.30	0.73
AC924	1	0.15	1.35	0.20
It´08	1	0.22	3.15	0.69

**Fig. 5.**
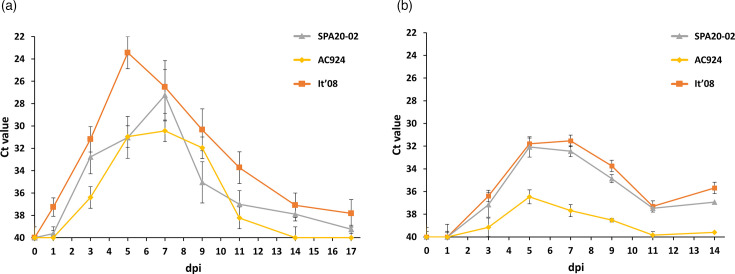
Viral genome load in feather pulps (**a**) and oral swabs (**b**) collected from the individuals of the three inoculated virus groups at different dpi. Bars represent the sem.

Viral genome was detected from 1 dpi to the end of the experiment in feather pulps from those groups infected with SPA20-02 and It´08. Meanwhile, in the group infected with AC924, viral genome in feathers was detected only from 3 dpi to 11 dpi in nine of the ten infected individuals (Fig. 5a). The group infected with It´08 showed the peak at 5 dpi with an average Ct value of 23.6. In SPA20-02- and AC924-inoculated groups, the peak was observed at 7 dpi, with average Ct values of 27.3 and 30.4, respectively (Fig. 5a). The group It´08 showed a significantly lower average Ct value in feathers throughout the experiment compared with the groups infected with AC924 (*P*<0.001) and SPA20-02 (*P*=0.03). No significant differences were observed in the whole viral-load curve in feathers between groups SPA20-02 and AC924 (*P*=0.07), although a lower average Ct value was found in SPA20-02-infected birds than in those inoculated with AC924 at 3 (*P*=0.03), 14 (*P*<0.01) and 17 dpi (*P*=0.02).

WNV genome was detected in oral swabs collected from the three inoculated groups from 3 dpi to the end of sampling (14 dpi), although viral loads were lower than those observed in blood and feathers. The peak of viral load in swabs was reached at 5 dpi in SPA20-02- and AC924-inoculated groups, with average Ct values of 32.1 and 36.5, respectively. The group inoculated with It´08 reached the peak of viral load in oral swabs at 7 dpi, with an average Ct value of 31.5 (Fig. 5b). The group infected with AC924 showed a significantly higher average Ct value in oral swabs throughout the experiment compared with the groups infected with SPA20-02 (*P*<0.01) and It´08 (*P*<0.01). There were no significant differences in the average Ct values in oral swabs throughout the experiment between the groups infected with SPA20-02 and It´08 (*P*=0.37).

All samples from the sham-inoculated group tested negative throughout the experiment. No viral RNA was detected in blood, oral swabs, feathers or organs by RT-PCR, and no infectious virus was recovered by plaque assay at any time point.

In euthanized individuals from the programmed necropsy groups, viral load was detected in the five organs analysed (brain, heart, kidney, liver and spleen) (Table 3). There were significant differences in the viral load between organs (*P*<0.01). We found significant differences between the liver and the heart (*P*<0.01), kidney (*P*=0.01) and spleen (*P*=0.01). Viral loads in organs from AC924-infected individuals were significantly lower than in organs from those infected with SPA20-02 (*P*<0.01) and It´08 (*P*<0.01). However, there were no significant differences in the viral load in organs between individuals infected with SPA20-02 and It´08 (*P*=0.40). Animals that succumbed to the infection showed viral load in all analysed organs. At the end of the experiment (17 dpi), only some birds, and in a limited number of organs, showed WNV load (Table 3). The three control birds necropsied at the end of the experiment did not show viral genome in any of their organs.

**Table 3.** Virus genome in organs (mean Ct values) by dpi and virus strain Ct values ≥40 were considered negative. The number of positive samples is indicated in brackets.

Isolate	Dpi	*n*	Brain	Heart	Kidney	Liver	Spleen
SPA20-02	3^†^	3	28.99 (3)	28.26 (3)	29.51 (3)	30.49 (3)	29.22 (3)
	6^†^	3	35.43 (3)	25.68 (3)	30.36 (3)	35.72 (2)	30.69 (3)
	7*	1	25.72	18.67	24.62	32.16	26.05
	9*	1	28.57	28.30	30.41	38.83	32.47
	10^†^	3	34.16 (3)	29.44 (3)	29.05 (3)	36.75 (2)	31.82 (3)
	17	5	36.34 (2)	39.02 (2)	33.17 (2)	Neg	37.18 (3)
AC924	3^†^	3	38.15 (2)	34.37 (3)	35.68 (3)	37.64 (3)	32.52 (3)
	6^†^	3	34.48 (1)	30.87 (2)	33.84 (2)	Neg	36.39 (2)
	10^†^	3	35.42	33.33 (3)	36.56 (2)	37.9 (1)	36.38 (3)
	17	5	38.44 (1)	Neg	Neg	Neg	39.72 (1)
It´08	3^†^	3	34.35 (3)	26.87 (3)	28.71 (3)	30.14 (3)	26.21 (3)
	4*^†^	1	28.70	16.46	17.36	16.42	13.25
	6^†^	3	31.44 (3)	23.65 (3)	27.60 (3)	33.75 (3)	30.07 (3)
	6*	1	18.22	21.84	18.37	20.51	25.62
	10^†^	2	31.71 (2)	35.17 (2)	28.71 (2)	39.97 (1)	32.62 (2)
	17	5	32.76 (3)	36.33 (3)	31.31 (1)	Neg	36.90 (5)

*Lethally infected partridges.

†Programmed necropsy groups.

Neg, negative.

### Seroconversion

**Fig. 6.**
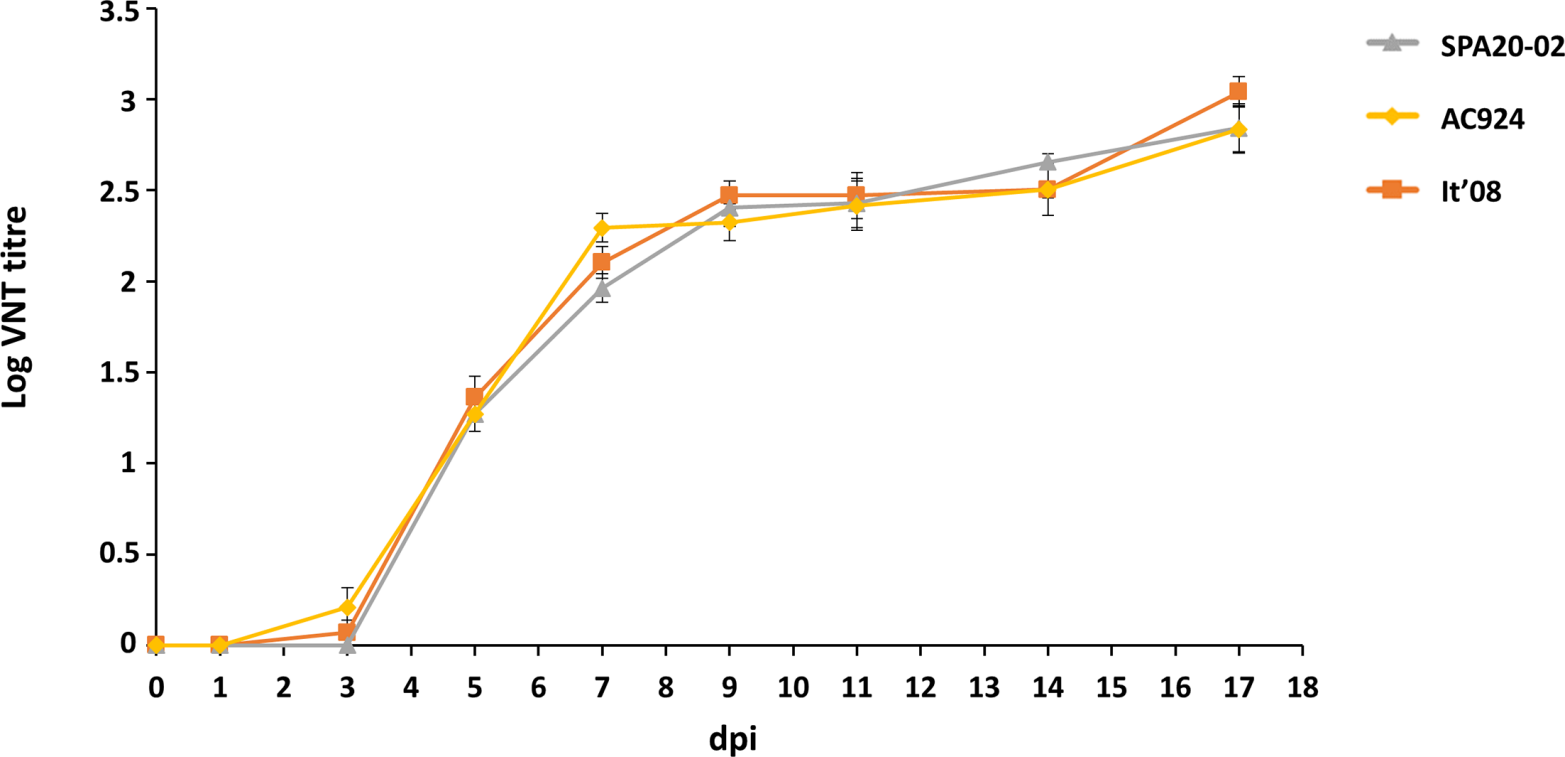
Neutralizing antibody response in the serum of inoculated red-legged partridges, measured by VNT. Bars represent the sem.

All surviving partridges inoculated with WNV developed neutralizing antibodies, and no differences were observed in log VNT titres between individuals infected with the different WNV strains (χ²=0.79; d.f.=2; *P*=0.67), including the individual infected with AC924 who did not show viraemia during the infection course. Neutralizing antibodies were first observed at 3 dpi in one individual infected with It´08 and in three individuals infected with AC924, while the first neutralizing antibodies detected in individuals infected with SPA20-02 were observed at 5 dpi. By day 5 after inoculation, all individuals had seroconverted, reaching maximum neutralizing antibody titres at 17 dpi (Fig. 6). The sham-inoculated group remained antibody-negative throughout the whole experiment.

## Discussion

The striking difference in severity observed between WNV outbreaks occurring in 2020 in south-western (Andalusia and Extremadura) and north-eastern (Catalonia) Spain deserves a detailed examination of the factors driving these disparities. These factors may involve extrinsic elements, such as the environmental conditions prevailing in each transmission setting, as well as intrinsic characteristics of the viral strains involved. Strain-related factors include pathogenicity and transmissibility. More pathogenic strains could cause increased morbidity and mortality per infection, and more transmissible strains may result in a greater number of infections and wider spread, even if their virulence remains unchanged.

In this study, we focused on intrinsic viral factors that may account for the observed differences in WNV dynamics. Specifically, we assessed the pathogenicity and host competence for transmission of two WNV strains circulating in Spain in 2020: the L1 strain SPA20-02 from Andalusia and the L2 strain AC924 from Catalonia, considered representatives of the respective epidemiological contexts. To this end, we conducted an *in vivo* experimental infection assay using red-legged partridges, an avian species previously shown to be susceptible to infection and disease, as well as transmission-competent hosts for different L1 and L2 WNV strains [42,48]. This species therefore provides a valuable model to evaluate the course of WNV infection, including pathogenicity and transmission capacity (host Ci), allowing for comparisons between different WNV strains of interest.

Our results confirmed that red-legged partridges are susceptible to infection and are competent hosts for the two strains examined. Based on the observed Ci, red-legged partridges showed higher host competence for SPA20-02 than for AC924.

Moreover, slightly higher morbidity was observed in the SPA20-02-inoculated group, which produced slightly higher mortality and a higher number of macroscopic lesions in organs compared with the AC924 strain (although these differences were not statistically significant). During the infection, viral-load profiles in blood, organs, feathers and oropharyngeal swabs were significantly higher in individuals infected with the SPA20-02 strain than in those infected with the AC924 strain.

Overall, the infection of the red-legged partridges with the SPA20-02 strain resulted in more efficient viral amplification in blood than infection with the AC924 strain, causing higher and more prolonged viraemia, and, as a result, leading to a higher transmission potential to mosquitoes. In the enzootic cycle, an increase in infected vectors implies a higher spread of the virus. Consequently, the results obtained in this study, indicating a lower host competence of avian reservoirs for north-eastern isolates, could partially explain the lower incidence of the disease in humans and horses observed in Catalonia in 2020 compared with the outbreaks in Andalusia and Extremadura in the same year, which were caused by south-western Spain L1 isolates such as SPA20-02.

In birds, comparative studies of L1 and L2 strains are limited. In experimental infections of house sparrows with a Central European L2 strain and a pathogenic L1 strain, no clinical signs were observed, although higher viraemia was detected for the L1 strain [36]. Nevertheless, other experimental assays reported similar pathogenicity and viraemia in red-legged partridges and falcons inoculated with L2 and certain highly pathogenic L1 strains [48,56]. The fact that both lineages have a range of strains showing different virulence phenotypes has been observed after experimental infections in mice, where different L2 strains showed virulence ranging from moderate to high, with some of them causing neuropathogenicity similar to that induced by highly pathogenic L1 strains [57]. Consequently, pathogenicity should be determined on a strain-by-strain basis, irrespective of the lineage to which they belong.

Despite the relatively high host competence observed in birds inoculated with the SPA20-02 strain (similar to that of the control strain used in this study, It´08, previously characterized as highly virulent), it did not exceed the Ci reported in the same species infected with other strains, including Spanish isolates obtained in years without human and equine outbreaks [40,42, 48]. Therefore, the competence of avian reservoirs for new L1 strains observed in this work seems does not seem to be the only cause of the high severity of the Andalusian outbreak in 2020. Hence, other intrinsic viral factors (such as differences in pathogenicity in mammals and vector competence), as well as extrinsic factors (including environmental conditions, weather, water availability, vector abundance, etc.), likely contributed to the epidemiological outcome.

Complementary to this study, assays on mice infected with SPA20-02, AC924 and other related strains revealed higher virulence in strains circulating in south-western Spain than in those circulating in the north-eastern part of the country (own unpublished data), which supports and complements, in part, the observations made in this study.

The 2020 outbreak in Andalusia coincided with an unusual increase in *Culex perexiguus* abundance in peri-urban areas, potentially enhancing virus circulation in birds and spillover to humans. Additionally, an increase in WNV infection rate in this mosquito population preceded the first human cases [27]. While *C. perexiguus* may have maintained enzootic cycles, *C. pipiens* could have acted as a bridge vector, infecting equids and humans.

Consequently, differences in outbreak severity between south-western and north-eastern Spain seem to be caused by several factors with additive effects, including host competence in birds, intrinsic pathogenicity in mammals and vector abundance.

Analysis of viral strains in this study focused on host competence, but data on vector competence are equally crucial for determining WNV transmission. Such investigations are essential to better understand viral transmission dynamics, in addition to the determination of host competence. Notably, *C. pipiens* shows no significant differences in transmission efficiency across L1 and L2 strains [58,59], whereas the role of *C. perexiguus* remains poorly characterized. Future studies should include vector competence assays for emerging WNV strains in both mosquito species to complement host competence analyses and to build a more comprehensive view of WNV outbreak dynamics.

In addition, future studies could include seroprevalence surveys in human populations from different regions of Spain to assess potential regional differences in exposure to WNV. These data would provide valuable insights into the intensity and persistence of viral circulation across areas with distinct epidemiological patterns, complementing both host and vector competence studies and contributing to a more integrated understanding of WNV transmission dynamics in the country.

The contrasting severity of the 2020 WNV outbreaks in different regions of Spain appears to be influenced, at least in part, by intrinsic viral factors, particularly differences in host competence between strains. Our findings confirm that the SPA20-02 strain circulating in Andalusia had a greater potential for amplification and transmission in avian hosts than the AC924 strain from Catalonia. However, this factor alone does not fully determine the magnitude of the outbreak. Other elements, including the abundance and distribution of competent mosquito vectors, their transmission capacity and the virulence of circulating strains in mammals, could have played an important role. Further integrative studies addressing both host and vector components are needed to better understand the complex dynamics underlying WNV outbreaks.

## Abbreviations

BSL-3: biosafety level 3
Ci: competence index
Ct: cycle threshold
d.f.: degrees of freedom
dpi: days post-inoculation
L1: lineage 1
L2: lineage 2
RT-PCR: reverse transcription polymerase chain reaction
VNT: virus neutralization test
WNF: West Nile fever
WNV: West Nile virus
